# Infarct volume as a predictor and therapeutic target in post-stroke cognitive impairment

**DOI:** 10.3389/fmed.2025.1519538

**Published:** 2025-02-04

**Authors:** Lingjia Xu, Dan Shan, Danling Wu

**Affiliations:** ^1^Department of Neurology, Shaoxing Second Hospital, Shaoxing, Zhejiang, China; ^2^Department of Biobehavioral Sciences, Columbia University, New York, NY, United States

**Keywords:** infarct volume, cognitive impairment, post-ischemic stroke cognitive impairment, cerebral infarction, ischemic stroke

## Abstract

Post-stroke cognitive impairment is one of the most common consequences of stroke, affecting more than half of stroke patients, especially in the geriatric population. Post-ischemic stroke cognitive impairment (PISCI) is particularly detrimental, as it can exacerbate a patient’s disability. Given that the severe consequences of adverse life outcomes are major contributors to disability and death among survivors of ischemic stroke, preventing stroke and PISCI remains a fundamental strategy for maintaining optimal brain health. Recent studies have extensively investigated the epidemiology, diagnosis, and management of PISCI. Nevertheless, significant gaps persist in our understanding of its pathophysiological mechanisms and potential therapeutic targets, which warrants further research. Factors such as baseline brain health, cerebral small vessel disease, and stroke characteristics (e.g., infarct location, severity, and morphology) have been associated with PISCI. However, its pathophysiology remains inadequately understood. Recent research suggests that infarct volume may serve as a novel indicator for predicting and managing PISCI. Thus, this review aims to expand our understanding of factors influencing PISCI and to elucidate its pathophysiological mechanisms. In particular, infarct volume has been proposed as a potential target and may play a critical role in predicting and managing PISCI. We advocate for improved and timely predictions of PISCI to enhance the quality of life for patients and reduce the economic and emotional burden on caregivers.

## Introduction

Ischemic stroke is a major cause of disability and mortality worldwide (1). The prevalence of post-ischemic stroke cognitive impairment (PISCI) has been extensively studied, but reported rates vary significantly due to differences in the applicability of cognitive assessment tools and the heterogeneity of study populations (2). A seminal meta-analysis estimated the prevalence of PISCI to be 53%, with approximately two-thirds of cases involving mild cognitive impairment and one-third classified as dementia (3). Notably, the prevalence in hospitalized patients, estimated at around 50%, may be underestimated, as 4–25% of patients deemed unevaluable remain at high risk for developing PISCI (4–6). An increasing number of research projects and clinical trials are now focused on enhancing acute-phase treatment for ischemic stroke (7–9). However, it is important to note that post-stroke complications continue to be the primary contributors to post-stroke morbidity and mortality on a global scale. PISCI is a common consequence of stroke that directly affects the patient’s function and quality of life and places a heavy burden on caregivers and healthcare systems. The occurrence and development of PISCI are influenced by multiple factors, including modifiable and unmodifiable risk factors, comorbidities, stroke characteristics, baseline brain health, and other elements (10, 11). As a result, early diagnosis, precise therapy, and comprehensive management of PISCI have become central research priorities.

The potential pathogenesis of PISCI is complex, with stroke characteristics such as severity, location, morphology, and a history of prior strokes being closely associated with its development. The significance of infarct location in PISCI is highlighted by lesions in areas critical for cognitive function processing in cerebral infarctions (12). Recent studies have shown a strong correlation between PISCI and infarctions in the left frontotemporal and thalamic regions, as well as the right parietal area (13). Morevoer, a notable link exists between PISCI and vertebrobasilar artery stenosis in the posterior circulation, likely resulting from insufficient perfusion to the hippocampus and posterior cingulate cortex (14). However, infarct location alone may not suffice to predict PISCI accurately, prompting researchers to suggest more comprehensive approaches, such as combining infarct volume with lesion location and lesion network mapping, to enhance prediction capabilities. Overall, the potential important role of infarct volume in PISCI has not been fully explored. Infarct volume may influence overall brain health and cognitive function, representing a key area for future research in the prediction and management of PISCI.

## Definition and influencing factors of PISCI

Post-ischemic stroke cognitive impairment is defined as encompassing all cognitive impairments that arise following an ischemic stroke, including both mild cognitive impairment and dementia (15). These impairments involve not only deficits localized to the site of the stroke lesion, such as aphasia or memory dysfunction, but also pre-existing cognitive impairments exacerbated by the stroke (16). The temporal pattern of PISCI is highly variable, with the most common presentation being cognitive impairment occurring within 3–6 months post-stroke. While cognitive impairment can be reversible during the early post-stroke period, up to one-third of these patients progress to dementia within 5 years (17). The current classification of PISCI distinguishes between early cognitive deficits, identified immediately after the stroke, and late cognitive deficits, which emerge in the subsequent months (18).

Post-ischemic stroke cognitive impairment can be influenced by various factors, including baseline health status (particularly cognitive state), diagnostic criteria, demographics, timing of assessment (19), and vascular risk factors (20). For instance, according to the REGARDS (Reasons for Geographic and Racial Differences in Stroke) Study, advancing age is significantly associated with accelerated cognitive decline following a stroke. For each 1 year increase in baseline age, the risk of cognitive impairment at annual follow-up increased by 17% (21). Additionally, stroke survivors with higher educational attainment may better compensate for vascular brain injury, thereby reducing their risk of PISCI.

Among individual vascular risk factors, diabetes and atrial fibrillation have been identified as strong predictors of PISCI. The CogFAST (Cognitive Function After Stroke) study reported that elderly stroke survivors with three or more vascular risk factors had a 3.6-fold increased risk of developing dementia post-stroke (22).

Cerebral small vessel disease (CSVD) also plays a critical role as a predictor of dementia in stroke patients. Imaging markers of CSVD, such as white matter hyperintensities, lacunar infarctions, cerebral microbleeds, cerebral atrophy, and enlarged perivascular spaces, independently or synergistically contribute to the development of PISCI (23). The cumulative burden of CSVD exacerbates the progression of PISCI by affecting multiple brain regions and compromising the integrity of specific white matter tracts. Notably, the preclinical phase of vascular cognitive impairment linked to CSVD often involves white matter integrity loss (24). Certain cognitive domains, including attention, executive function, processing speed, and language abilities, have been correlated with the extent of white matter lesions in stroke patients (25). Overall, optimizing the management of stroke-related factors and vascular risk profiles may help mitigate the risk of PISCI.

Post-ischemic stroke cognitive impairment is also associated with other post-stroke complications (26). For example, post-stroke delirium is a common clinical phenomenon that many researchers consider indicative of pre-existing cognitive impairment. It has been associated with an increased risk of cognitive decline following a stroke. However, the relationship between post-stroke delirium and PISCI remains unclear, as delirium may interfere with accurate cognitive assessments (27). Additionally, post-stroke depression significantly impacts patients’ independence and hinders their reintegration into community roles, further compounding the burden of stroke-related complications (28). Studies have found similarities in the network patterns between depression and cognitive performance, with psychomotor function and attention being key components connecting depression and cognition (29). Symptoms associated with post-stroke depressive apathy (e.g., exhaustion, dysesthesia) were significantly associated with greater impairment of executive function, memory, and overall cognitive function, implying that patients with cognitive impairment may benefit from interventions for post-stroke depression (30, 31).

## Potential pathogenesis of PISCI

### Stroke characteristics

Several stroke characteristics serve as predictors of post-stroke cognitive decline or dementia, including stroke severity, infarct location, number of infarctions, and infarct morphology. For instance, one study reported that severe strokes preceded the onset of dementia by an average of 25 years, whereas mild strokes preceded dementia onset by approximately 4 years (32, 33). Additionally, an interaction appears to exist between the number and morphology of infarcts and the development of PISCI (34). Multiple infarcts have long been recognized as a cause of vascular dementia, which typically presents as a gradual or fluctuating decline in cognitive function. A pooled analysis of existing data revealed that multiple strokes were associated with a 2.8-fold increased risk of developing post-stroke dementia (32).

The location of the cerebral infarction plays a crucial role in the manifestation of PISCI symptoms (12). The notion that strategic infarction (i.e., infarction located in a key area of cognitive processing) leads to vascular dementia remains controversial, but most researchers agree that lesion location plays a pivotal role in the development of PISCI. Strategic locations such as the left frontotemporal lobe, left angular gyrus, left basal ganglia and surrounding white matter, left thalamus, right parietal lobe, and areas supplied by the left middle cerebral artery are associated with a heightened risk of PISCI (12, 35). Nevertheless, cognitive impairments in patients with aphasia might be overestimated due to the overlap of these areas with language functions, which are integral to cognitive assessments (17). While infarct location alone may not fully predict cognitive dysfunction, it is often correlated with specific neurological abnormalities. Its impact on cognitive function may be synergistic, particularly when combined with factors such as infarct volume and the burden of cerebral small vessel disease.

Moreover, PISCI is influenced by the nature and extent of brain injury associated with specific stroke subtypes, as well as by long-term, multi-stage diffuse brain injury or the effects of acute stroke interventions (36). Common cognitive deficits in PISCI include problems with executive function and attention, although studies also report widespread deficits across multiple cognitive domains (37, 38). Similar patterns of cognitive deficits are observed in patients with intracerebral hemorrhage and acute ischemic stroke (17, 39), with executive function and verbal memory being particularly impacted in those with subarachnoid hemorrhage (40).

### Brain health status

The predominant pathogenesis of cognitive decline and dementia following cerebral infarction is largely attributed to cerebral small vessel disease (CSVD) (19). CSVD is also an important indicator of brain health, though the precise pathophysiology of PISCI remains elusive. Factors such as brain reserve and resilience are critical for brain health. Brain reserve reflects the disparity between the extent of brain damage and its clinical manifestations (41), while brain resilience refers to the brain’s capacity to withstand cumulative damage, with compensatory mechanisms mitigating its effects (42, 43).

The neurovascular unit (NVU) plays a central role in brain health, reserve, and resilience at the cellular level. The NVU is the smallest functional component of brain tissue, consisting of neurons, glia, and vascular cells (44). It is essential for controlling cerebral blood flow and preserving the integrity of the brain’s parenchymal environment (45). Recent discussions on “brain health” suggest that the NVU’s ability to withstand the impacts of metabolic diseases, acute inflammation, and cerebrovascular injury is key (46). Conversely, damage to the NVU can compromise brain health, leading to stroke, dementia, and other neurological diseases (47), and its integrity is crucial for promoting optimal brain health.

The mechanism by which NVU dysfunction contributes to PISCI is multifaceted. First, vascular risk factors lead to microvascular dysfunction, disrupting the blood-brain barrier, impairing clearance, and allowing neurotoxic molecules to invade the brain (46, 48). Second, neuronal injury and neurodegeneration are accelerated by the buildup of neurotoxic plasma proteins and a decrease in cerebral blood flow. Third, dysregulation of the NVU may enhance the production of amyloid beta (Aβ) and slow its clearance, contributing to Aβ accumulation (49, 50). This synergistic effect is believed to contribute to the onset of dementia and cognitive impairment.

### Pathological synergistic effects and genetic contribution

Given the shared risk factors among stroke, dementia, and PISCI, neuropathology associated with cerebrovascular disease may accelerate the onset of PISCI (51). For instance, individuals with mixed neuropathological findings associated with vascular dementia (VD) and Alzheimer’s disease (AD) have a threefold higher likelihood of more rapid disease progression compared to those with only one type of neuropathological finding (52–54). Stroke or CSVD patients with evidence of Aβ deposition experience more rapid cognitive decline than those without Aβ pathology (55). Groundbreaking studies have revealed that approximately one-third of PISCI cases are associated with AD, with this relationship further clarified by amyloid positron emission tomography studies (56–58). These findings suggest that preclinical AD significantly increases the risk of PISCI. Additionally, cerebral amyloid angiopathy (CAA), an independent factor associated with cognitive impairment in AD, has been linked to an increased risk of PISCI through MRI markers (59). This suggests that CAA may also contribute to PISCI, warranting further investigation into the role of comorbid proteinopathies in PISCI pathogenesis.

Genome-wide association studies (GWAS) of stroke and dementia families provide important insights into their genetic basis of these conditions (60). Given the interactions and synergies between VD and AD, genetic factors involved in amyloid production or elimination pathways may confer susceptibility to dementia following vascular brain injury (61). A meta-analysis of genetic polymorphisms identified five polymorphisms associated with VD, including the Apolipoprotein E (APOE) ε4 allele (62). The APOE ε4 allele is the strongest genetic risk factor for late-onset AD (60) and is also associated with CSVD markers and CAA (63). However, the relationship between APOE and PISCI is unclear. Stroke survivors homozygous for APOE ε4 have a 2.9-fold increased risk of developing dementia over 5 years compared to those homozygous for ε3 (64). Other genetic variants, such as those in the genes encoding angiotensin-converting enzyme and endothelial nitric oxide synthase, have been linked to dementia events in VD and elderly stroke survivors (65, 66). Genetic screening of stroke survivors may aid early diagnosis of PISCI, and further research is needed to explore the genetics of PISCI.

## Infarct volume as a new indicator for PISCI

### Infarct volume and prognosis of ischemic stroke/cerebral infarction

Cerebral infarction results from ischemia and hypoxic necrosis of brain cells due to insufficient brain tissue perfusion (67). The outcomes and prognoses of cerebral infarcts vary significantly depending on their degree and location, underscoring the importance of precise characterization. Pathologically, brain tissue damage in cerebrovascular disease is three-dimensional, making volume-based descriptions of injury and necrosis areas more accurate, intuitive, and realistic. Furthermore, many large cohort studies on reperfusion therapy for ischemic stroke also select participants based on infarct core volume (68, 69), such as the Endovascular Therapy Following Imaging Evaluation for Ischemic Stroke 3 (DEFUSE3), which uses a threshold of 70 mL to define the infarct core volume for case selection (69). It has been found that in acute ischemic stroke, the larger the volume of irreversible damage, the more severe and permanent the clinical defect (70).

Although infarct volume is often used as a predictor of clinical outcomes in acute ischemic stroke and as a surrogate outcome in several studies, this relationship may not always be linear (71). Ospel et al. (72) explored the relationship between infarct volume and clinical outcome, hypothesizing that the relationship varies depending on infarct size. Their findings demonstrated that larger infarct volumes in acute ischemic stroke patients correlate with more severe functional loss, making infarct volume a strong predictor of clinical prognosis. For small infarcts, infarct volume does not reliably predict clinical outcomes. However, for moderate to large infarcts, a linear relationship exists between larger infarct volumes and a lower likelihood of favorable outcomes. For very large infarctions, adverse outcomes are almost certain.

These findings suggest that lesion mapping, aimed at understanding structure-function relationships, may be more beneficial for smaller infarcts. However, infarct volume, as a significant marker for predicting the prognosis of cerebral infarction stroke, serves as a useful surrogate index within a certain range (72, 73).

### Relationship between infarct volume and PISCI

The relationship between infarct volume and PISCI should be examined in the context of infarct location, as the location of cerebral infarction is a key determinant of PISCI. The association between infarct location and cognitive outcomes has been extensively studied, highlighting its potential role in the development of PISCI (12). Increasing research utilizes lesion-symptom mapping approaches to elucidate the neuroanatomical bases of specific cognitive processes (74). A study has suggested that the predictive value of cerebral infarction location for PISCI is significant. However, lesion location is inherently linked to the cause of stroke as well as to lesion size and volume. PISCI is particularly associated with infarctions in the left frontotemporal lobe, left thalamus, and right parietal lobe (12). Different cognitive impairments correlate with various infarct locations (75). The etiology of the stroke, along with the extent and size of the lesion, are closely associated with PISCI and are directly related to the lesion’s location. For instance, small subcortical infarcts in supratentorial areas differ in distribution from cortical and larger subcortical infarcts (32). Specifically, the left thalamus is a predictor of PISCI in stroke subtypes involving macrovascular or small vessel lesions, whereas other subcortical regions become predictive only after larger cerebral infarctions (12).

A large infarct volume is also a significant risk factor for cognitive impairment. A study from Indonesia found that patients with PISCI had higher infarct volumes compared to those without PISCI, with vascular risk factors, the location of the infarct, and the severity of the stroke showing no differences between the groups (76). In line with the findings of Liang et al.’s study (77), the multivariate logistic regression revealed that patients with greater infarct volumes (≥0.054 ml) were more likely to experience PISCI (76). These insights enhance our understanding of the relationship between infarct volume and PISCI, aiding in its early prediction and the formulation of better prevention strategies.

In a study of subclinical cerebral infarction following carotid artery intervention, embolic infarct volume was found to correlate with cognitive function measured by Rey Auditory Verbal Learning Test (RAVLT) (78). There was an overall trend of improvement in RAVLT scores after carotid revascularization, and a significant increase in infarct volume was observed in patients with decreased RAVLT, further research also suggested that the volume of embolic infarcts was associated with long-term cognitive changes. Myers et al. (79) evaluated the effect of acute minocycline treatment after stroke on reducing infarct volume and the expression of chronic microglia and astrocytes in distal white matter regions, as well as its beneficial effects on various domains of cognitive function after stroke. Mangin et al. (80) demonstrated that immunomodulatory drugs could reduce infarct volume and pro-inflammatory mediators, enhance early neurogenesis, accelerate sensorimotor recovery, and prevent long-term memory loss in diabetic mice. Another study on the relationship between acute infarct volume and health-related quality of life (HRQOL) after ischemic stroke, which evaluated domain-specific quality of life scores for acute cerebral infarction at 3 months after stroke, included a total of 490 patients, and found that infarct volume was associated with poor prognosis, but more cognitive-related evaluations are needed because they only focus on general cognitive concerns (81).

**FIGURE 1:**
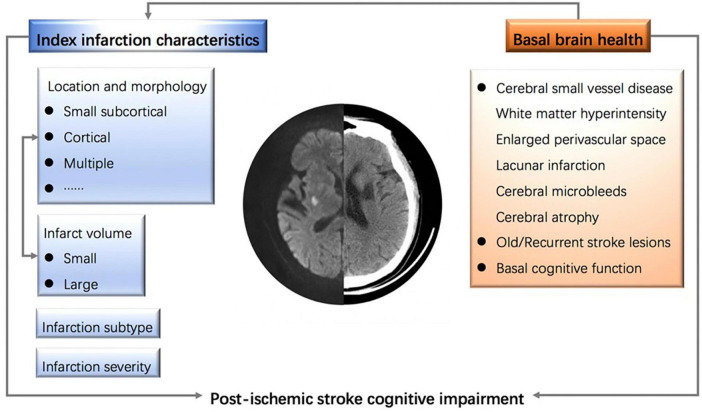
Influencing factors of post-ischemic stroke cognitive impairment.

Additionally, the clinical history, burden of cerebral small vessel diseases, and the interplay between infarct locations are confounding factors that may collectively influence a patient’s cognitive function (73). It is important to note that predicting PISCI does not necessarily imply causality. Attention should also be given to the dynamic changes in infarct volume during the acute and subacute phases of stroke, including the progression and regression of cerebral edema. Over time, the characteristics of a cerebral infarction on CT/MRI scans evolve as the lesions progressively become less dense and more distinctly contoured, which may impact the accurate assessment of infarct volume. Figure 1 summarizes the influencing factors of PISCI.

Infarct volume may serve as a novel prognostic indicator for PISCI following cerebral infarction. It is essential to recognize that larger infarct areas often encompass critical neurological regions and cognitive functions, suggesting a higher likelihood of cognitive deficits. Given the intricate relationship between brain structure and function, even minor lesions in strategic locations, such as an anterior choroidal infarction, a small but clinically significant site, may lead to severe disabilities (82).

However, the ability to reliably predict clinical outcomes based on infarct volume can be limited at an individual level (70). Treatment approaches for patients with minor infarcts could be substantially affected by the involvement of specific critical anatomical areas. Additionally, the prognosis for small-volume infarctions may depend not only on the infarct itself but also on subsequent complications (83), underscoring the importance of continuous stroke care and the prevention of stroke-related complications. Prompt, high-quality reperfusion remains crucial to minimizing infarct volume.

## Discussion

Although the pathophysiological processes underlying PISCI are complex and influenced by numerous factors, it has garnered significant attention as a major condition affecting brain health in the elderly. This review examines the definition, influencing factors, potential pathophysiological mechanisms, and recent research advances related to PISCI. Additionally, it explores infarct volume as a potential novel indicator that may enhance the prediction and management of PISCI.

Attention should also be given to the dynamic nature of PISCI, where cognitive performance can fluctuate due to compensatory repairs, secondary neurodegeneration, and recurring cerebrovascular events (19). The long-term trajectory of cognitive function following a stroke remains uncertain. A study that examines long-term cognitive alterations following a stroke was published on the Stroke and Cognition Consortium (84). A turning point was found approximately a year after the stroke, with those suffering from initial ischemic stroke exhibiting a brief but significant improvement at first, followed by a decline starting a year later. Similar rates of cognitive change were noted in both the overall and specific cognitive domains, with the exception of executive function.

Research on the longitudinal relationship between stroke severity and cognitive decline is sparse (85, 86). Future studies should more comprehensively assess the longitudinal changes in infarct severity and cognitive function, beginning before the stroke and extending through long-term follow-up. This evaluation should explore how both the location and subtype of cerebral infarction influence resultant cognitive impairments. Understanding the influence of infarction size, location, and volume on severe stroke outcomes and cognitive recovery is crucial. The variation in the risk of cognitive impairment by stroke subtype is not well understood (87), and more studies are needed to evaluate cognitive outcomes across different stroke subtypes in both short- and long-term scenarios.

The influencing factors of PISCI are diverse and multifaceted, with its specific pathogenesis remaining unresolved. Furthermore, significant challenges persist in elucidating the correlation between infarct volume and PISCI. Patients’ cognitive symptoms and prognosis are related to the complex interplay between infarct volume and location, the subtype and severity of infarction, the effect of additional problems following infarction, and the dynamic changes in cognitive function. Multi-subgroup, multicenter investigations examining cognitive outcomes in relation to infarct location and volume, particularly through long-term follow-up studies of longitudinal relationships, represent promising approaches. Such studies are especially relevant in the post-pandemic era, where the synergistic impact of COVID-19 infection further elevates the risks of cognitive impairment and potential new-onset dementia (54).

## Conclusion

Post-ischemic stroke cognitive impairment significantly hinders the recovery process in patients with cerebral infarction. It is crucial to focus on stroke-related mental health rehabilitation, alongside rehabilitation for limb muscle strength, language functions, swallowing, and others (2, 88). More research is needed to improve the identification and timely intervention of PISCI. Although PISCI is influenced by the location of the cerebral infarction, infarct volume may serve as a novel predictor. However, given the current state of research, a nuanced analysis of its relationship with PISCI remains necessary. Future studies should focus on exploring infarct volume as a new therapeutic target for PISCI, such as strategies to reduce infarct volume (89), and utilizing it for risk stratification to develop a PISCI prediction model. This approach could potentially reduce the onset and progression of PISCI, improve patient outcomes, and decrease the public health risk.
